# Infectious Endocarditis from* Enterococcus faecalis* Associated with Tubular Adenoma of the Sigmoid Colon

**DOI:** 10.1155/2017/3095031

**Published:** 2017-08-07

**Authors:** Emilly Caroline de Freitas Silva, Camila Ronchini Montalvão, Simone Bonafé

**Affiliations:** Centro de Ensino Superior de Maringá (UNICESUMAR), Avenida Guedner, 1610 Jardim Aclimação, 87050-390 Maringá, PR, Brazil

## Abstract

**Introduction:**

* Enterococcus faecalis (E. faecalis)*, a constituent of the gut microbiota, can be associated with both colonic lesions and endocarditis. Since this microorganism is one of the endocarditis etiological agents, there is a need for greater study in regard to the association with endocarditis and colonic lesions.

**Case Presentation:**

This is the case description of a 53-year-old man with history of prolapse of the anterior mitral valve leaflet who was diagnosed with endocarditis by* E. faecalis* and treated with ampicillin and gentamicin. Upon investigation by colonoscopy, he was found to have a tubular adenoma with low grade dysplasia.

**Conclusion:**

There are a few descriptions in scientific literature of an association between endocarditis by* E. faecalis* and colonic lesions. However, further studies with significant correlation between the two pathologies are required, so that proper measures can be implemented in clinical practice.

## 1. Introduction

Little is known about the pathophysiology of the interactions between infective endocarditis caused by* E. faecalis* and intestinal neoplasia. A few descriptions in literature associate the presence of these bacteria with preexisting colonic lesions [1–5]. It is recognized and well established that there is a strong correlation between these two pathologies when the isolated bacteria are* Streptococcus gallolyticus (S. gallolyticus)*, formerly known as* Streptococcus bovis (S. bovis)* [6, 7].

In this context, the objective of this case study is to present clinical, diagnostic, and therapeutic data of a patient that developed endocarditis by* E. faecalis* with coinciding colonic lesions.

## 2. Case Presentation 

A 53-year-old male with history of prolapse and discrete myxomatous degeneration of the anterior mitral valve leaflet, mild-to-moderate aortic insufficiency, and midsystolic mitral regurgitation presented fever, nocturnal hyperhidrosis, myalgia, and anorexia during a period of approximately 15 days, without significant weight loss.

An investigation to elucidate the origin of the fever was initiated. The hemogram showed discrete leukocytosis (10^5^ cells/ml) and an increase of the inflammatory markers ESR (98 mm) and CRP (97 mm/L). Serologies for cytomegalovirus, Epstein Barr virus, HIV, toxoplasmosis, and syphilis were all negative, as well as ANA and PPD, which was nonreactive.

Urinalysis presented with intense leukocyturia (289.740 cells/ml) and urine culture showed the presence of* Escherichia coli*. After collecting 3 samples of blood for culture (BacT/Alert 3D, Biomerieux method), it was decided to start antibiotics for urinary tract infection (UTI), even though the patient had no UTI symptoms at the time. The patient denied being submitted to any invasive urinary or gastrointestinal tract procedures, like the placement of vesical catheters or surgery.

**Figure 1:**
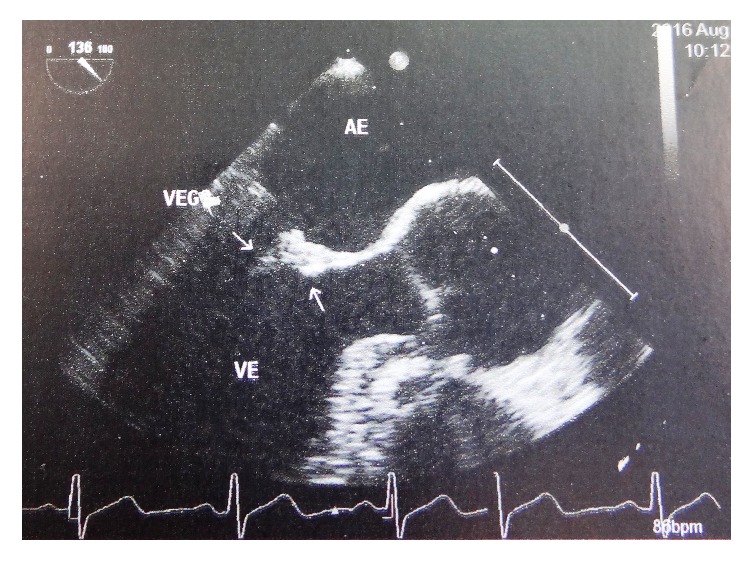
Vegetation on the anterior leaflet of the mitral valve shown on transesophageal echocardiogram.

Despite the use of gentamicin for the asymptomatic UTI, the initial symptoms persisted. Approximately 10 days after the initial investigation, the patient evolved with Janeway lesion and the onset of new diastolic aortic and systolic mitral murmurs. A transesophageal echocardiogram was performed and it revealed a discrete 5 mm mass (vegetation) on the anterior mitral valve leaflet (Figure 1). Furthermore, culture and antibiogram from all 3 previously collected blood samples revealed the presence of* E. faecalis* sensitive to ampicillin.

The patient was diagnosed with endocarditis and was subsequently hospitalized and prescribed ampicillin for a 28-day therapeutic cycle (due to a shortage of crystalline penicillin). Furthermore, gentamicin was extended for 14 days.

**Figure 2:**
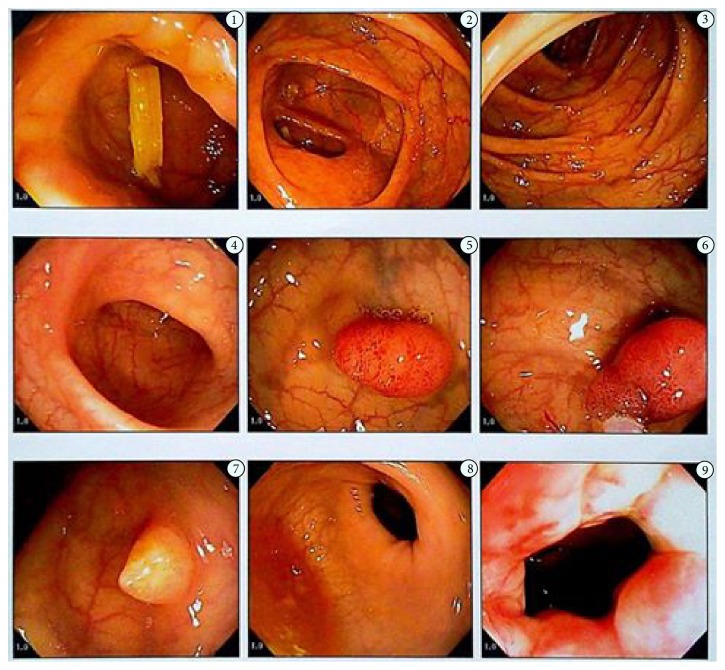
Images of videocolonoscopy that show the presence of polyps.

On the 23rd day of hospitalization a colonoscopy was solicited, given the patient's age, family history of colonic cancer, and scientific literature descriptions associating endocarditis with intestinal diseases. Intestinal preparation was duly performed and there was complete visualization of the colon up to the ileocecal valve (Figure 2). A 15 mm pedunculated polyp was visualized in the sigmoid colon and a polypectomy was performed. The final anatomopathological report determined tubular adenoma with low grade dysplasia.

The interval between the onset of symptoms and the conclusion of the case was approximately 2 months. The patient was released from the hospital following the end of the treatment with resolution of the infectious manifestations and, without complications, was referenced to follow-up cardiology care.

## 3. Discussion 

*Enterococcus* is the third major cause of infectious endocarditis in the world, being responsible for 5% to 15% of cases.* E. faecalis*, responsible for the majority of these cases, is a minor commensal bacteria found in the intestinal microbiota [8].

The source of bacteremia by* E. faecalis*, in cases with cardiac infection as well as alterations in other systems, most often is undetermined. Research shows that up to 67.8% of bacteremia cases do not have an identifiable source [9, 10]. A frequent point of entrance, through which the* E. faecalis* invades the bloodstream, is the genitourinary tract, particularly in cases related to endocarditis. Contaminations through the gastrointestinal and biliary tracts, as well as catheters, are also cited in various studies [1, 9].

Possible associations between endocarditis by* E. faecalis* and colonic lesions have been demonstrated [1–5], although the data described in studies is insufficient to determine the magnitude and the meaning of this combination. Such an association is recognized with* S. gallolyticus*, being extensively documented in publications, even though the pathophysiological mechanism is not yet well elucidated [11].

These studies use a new nomenclature for what was previously known as* S. bovis*,* S. bovis* biotype I, and biotype II/2, which now correspond to* S. gallolyticus* and its subspecies. These microorganisms are mentioned in studies as being associated with colorectal cancer and with the development of bacteremia and endocarditis [11].

The latest discoveries, through genomic analysis, showed that* S. gallolyticus* has intrinsic characteristics capable of favoring its translocation, such as proteins that bind to collagen and the presence of 3 types of pili. These same characteristics, which make this microorganism highly proficient in causing bacteremia, also have the potential to cause endocarditis and intestinal lesions [12].

Regarding* E. faecalis*, it is believed that this agent is also involved in the mutation of colonic cells [13]. Some studies defend that the lesioned mucosa, be it due to ischemia or inflammation, is propitious to translocation of agents such as* E. faecalis*, leading to bacteremia [2, 9, 14]. It is known that the presence of a previous mitral prolapse predisposes to the onset of endocarditis caused by this microorganism.

Recent studies about inflammatory intestinal diseases and irritable bowel syndrome have pointed to potential mechanisms through which* E. faecalis* would be able to cause a dysfunction of the intestine's epithelial barrier [15]. However, even though some researchers have tried to show that in fact these bacteria found in the blood and in other sterile regions are originated from the gastrointestinal tract, there has been little progress and it has not yet been possible to comprehend how this species migrates [16]. Nonetheless, it is understood that the translocation of* E. faecalis* through healthy tissue is a rare event and has no damaging lasting repercussions [17, 18].

Regarding the case described in this study, the source of the infection was not identified. However, its simultaneity with a low grade intestinal dysplasia was observed, which can reinforce the hypothesis of* E. faecalis* being capable of translocating itself and causing endocarditis having, as a point of entry, a previous intestinal lesion. It is also relevant to emphasize that colorectal cancer, which originates from the same pathological process present in the patient being studied, is the fourth most prevalent neoplasia in the Brazilian male population, being relatively frequent in the age group of 50 years and older (INCA ESTIMATIVA 2016). Considering the fact that the patient here described is in the aforementioned gender and age group, such bias could be confounding in the sense of attributing the described mechanism to the endogenous intestinal* E. faecalis*. However, even with this limitation, this case description aggregates with others that have suggested the same supposed interaction [1–5].

## 4. Conclusion

The occurrence of endocarditis by* E. faecalis* of a nonidentifiable source is not uncommon, as seen with the frequency of cases in which there was a coexistence of intestinal disease and enterococcal endocarditis. Considering the inconclusive investigation of data so far, in order to be admissible to affirm a significant correlation between these two pathologies, it is important that further investigation with colonoscopy be performed in such cases, with the objective of elucidating the relevance of the association.
